# The association between periodontal disease and pancreatic cancer: epidemiology, mechanisms, and implications for clinical management

**DOI:** 10.3389/fcimb.2026.1817579

**Published:** 2026-04-27

**Authors:** Binbin Wang, Hongliang Cao, Gengchen Huang, Yifan Song, Yutao Ma, Zihan Gao, Shuxin Li, Zhijun Tang, Haiyang Zhang, Wei Wei

**Affiliations:** 1 Department of Urology II, The First Hospital of Jilin University, Changchun, China; 2 Key Laboratory of Pathobiology, Ministry of Education, Jilin University, Changchun, China; 3 Department of Prosthodontics, Hospital of Stomatology, Jilin University, Changchun, China

**Keywords:** chemoresistance, clinical implications, oral microbiota, pancreatic cancer, pathogenic mechanisms, periodontal disease, porphyromonas gingivalis

## Abstract

Pancreatic cancer (PC), particularly pancreatic ductal adenocarcinoma (PDAC), is a highly lethal malignant tumor with poor prognosis, limited early screening strategies, and high chemoresistance. Periodontal disease (PD), a prevalent chronic inflammatory disorder caused by oral microbiota dysbiosis, has been increasingly linked to systemic diseases, including cancer. This review summarizes the epidemiological evidence, potential pathogenic mechanisms, and clinical management implications of the association between PD and PC. Epidemiological studies, including meta-analyses, cohort studies, and case-control studies, consistently demonstrate that PD (especially moderate-to-severe periodontitis and combined gingivitis-periodontitis) is associated with an increased risk of PC incidence and mortality, with significant population heterogeneity (more prominent in middle-aged and elderly individuals, males, and those with comorbid diabetes). Specific periodontal pathogens, such as Porphyromonas gingivalis and Aggregatibacter actinomycetemcomitans, play key roles in this association, showing dose-response relationships and preceding PC onset. Mechanistically, PD contributes to PC development through multiple interconnected pathways: translocation and colonization of periodontal pathogens in pancreatic tissue, induction of precancerous lesions (e.g., acinar-to-ductal metaplasia), activation of inflammatory signaling pathways (TLR4/NF-κB, Wnt/β-catenin), immune dysregulation and tumor immune escape, microbiota imbalance and carcinogenic metabolite accumulation (e.g., acetaldehyde, nitrosamines), and induction of chemoresistance (via cytidine deaminase-mediated gemcitabine inactivation or Notch1 pathway activation). Clinically, this association provides novel perspectives for PC prevention and management: integrating periodontal health assessment into PC risk screening, developing non-invasive diagnostic biomarkers (oral microbiota, miRNAs, saliva metabolites), optimizing treatment strategies (targeted antibiotics, probiotics, phage therapy), and establishing a multidisciplinary collaboration (MDT) model involving dentistry, oncology, gastroenterology, and microbiology. In conclusion, PD is a potentially modifiable risk factor for PC, and oral health management may serve as a cost-effective strategy for PC prevention and improved prognosis. Future prospective studies are needed to confirm the causal relationship and standardize clinical practices for this cross-organ association.

## 1 Introduction

Pancreatic cancer (PC) is a malignant tumor originating from pancreatic ductal epithelial cells or acinar cells, among which pancreatic ductal adenocarcinoma (PDAC) accounts for over 90% and represents the most common clinical subtype. As one of the most lethal malignant tumors globally, PC poses a severe threat to human health (Zhang et al., 2023). According to the 2022 Global Cancer Statistics, new PC cases account for 2.6% of all new cancer cases worldwide, ranking 12th, while PC-related deaths account for 4.8% of total cancer deaths, ranking 6th. The significantly higher mortality rate than incidence reflects the inferior prognosis of this disease (Bray et al., 2024). PC is an age-related disease predominantly affecting middle-aged and elderly populations, with the risk of onset increasing significantly with age (Bray et al., 2024). More critically, its disease burden continues to grow. Currently, PC is the third leading cause of cancer-related deaths in the United States, and it is projected to become the second leading cause by 2030. By 2040, the incidence and mortality rates of PC worldwide are expected to rise by more than 75% and 80%, respectively (Stoffel et al., 2023; Hesami et al., 2025; Jamal and Khan, 2025; Stoop et al., 2025). Despite ongoing advances in scientific discovery and medical technology, the clinical diagnosis and treatment of PC remain extremely challenging. Currently, there are insufficient preventive measures and screening strategies for the general population, making early diagnosis difficult (Signoretti et al., 2017).

Additionally, early PC symptoms lack specificity; even when tumor cells have become highly invasive, the vast majority of patients remain asymptomatic during the early stages and the progression to advanced metastasis (Fan et al., 2018; Ungureanu et al., 2023). Only approximately 20% of patients are diagnosed at an early, surgically resectable stage (Klein, 2021). The current standard treatment for PC is surgical resection, supplemented by comprehensive therapies such as chemotherapy (e.g., gemcitabine) and radiotherapy. However, PDAC generally exhibits resistance to targeted therapy, immunotherapy, chemotherapy, and radiotherapy, further increasing the difficulty of treatment (Bear et al., 2020). Even after radical treatment, most patients experience recurrence, with a 5-year survival rate of only 2% to 9% (Zhao and Liu, 2020). To date, the etiology of PC has not been fully elucidated, and known risk factors such as smoking, obesity, and diabetes can only explain a subset of cases (Maisonneuve and Lowenfels, 2015). Recent studies have revealed that the pancreas is not a sterile organ, and the resident microbiota and the “pancreas-microbiota axis” may be involved in the pathogenesis of PC (Thomas and Jobin, 2020). Furthermore, chronic inflammation, KRAS oncogene activation, and microbiota-induced barrier disruption can synergistically promote carcinogenesis, suggesting that microbiota-related risk factors may represent a key breakthrough in research (Papa et al., 2023). Therefore, in-depth exploration of novel risk factors and pathogenic mechanisms is of great significance for improving the prognosis of PC.

Periodontal disease (PD) is a group of chronic inflammatory disorders triggered by oral microbiota dysbiosis, primarily affecting periodontal supporting tissues, including the gingiva, periodontal ligament, cementum, and alveolar bone. Its core subtypes include gingivitis and periodontitis (Bracci, 2017; Villar et al., 2024). The human oral cavity harbors a complex microbiome, with over 800 bacterial taxa cataloged and approximately 525 identified as primarily oral species in the latest update of the Human Oral Microbiome Database (HOMD Version 4.2; https://www.homd.org (Escapa et al., 2018)). Under normal physiological conditions, the microbiome maintains equilibrium with the host immune system. When this balance is disrupted, dental plaque accumulation can lead to gingivitis, characterized by gingival inflammation and bleeding. If left untreated, gingivitis progresses to periodontitis, characterized by periodontal pocket formation, gingival recession, and progressive destruction of connective tissue and alveolar bone, ultimately leading to tooth loss (Michaud et al., 2017; Akbari et al., 2024). Epidemiological data indicate that PD is highly prevalent, affecting up to 90% of the global population, with severe PD occurring in approximately 10% of individuals, making it the 11th most common health problem worldwide (Hao et al., 2019; Janakiram and Dye, 2020). Notably, PD is not merely a local oral lesion: its pathogenic bacteria and toxins can enter the systemic circulation through damaged epithelia, and gingival tissues under inflammatory conditions produce inflammatory mediators such as tumor necrosis factor-α (TNF-α) and interleukin-1β (IL-1β), which diffuse systemically and impact overall health (Pizzo et al., 2010). Studies have demonstrated a close bidirectional association between PD and various systemic diseases, including cardiovascular diseases, diabetes, rheumatoid arthritis, and respiratory diseases (Ungureanu et al., 2023; Yamazaki, 2023; Yamazaki and Kamada, 2024). The major pathogenic bacteria of PD include Porphyromonas gingivalis (P. gingivalis) and Tannerella forsythia (Chattopadhyay et al., 2023). Among these, P. gingivalis is detected in 50% to 80% of patients with severe periodontitis, a rate significantly higher than that in healthy individuals. Furthermore, the colonization rate of P. gingivalis in individuals aged 0–18 years can reach 37%, suggesting that microbial colonization may be established early in life (Zou et al., 2025). Additionally, poor oral health may alter the oral and gut microbiota, leading to excessive local immune responses and exacerbating systemic inflammation (Zhang et al., 2020).

In recent years, as research into the relationship between the microbiota and tumorigenesis has deepened, the association between PD and PC (PD-PC association) has emerged as a research hotspot. Populations with poor oral health have a significantly increased risk of developing PC, and alterations in oral microbiota composition may be a key driver (Liu et al., 2019). Critically, the presence of specific periodontal pathogens such as P. gingivalis in saliva several years before PC diagnosis is associated with a significantly elevated risk of PC (Fan et al., 2018). Moreover, established PC risk factors like smoking may contribute to disease development by modifying the oral microbiota (Thomas and Jobin, 2020). The discovery of this association not only provides a novel perspective on the etiology and prevention of PC but also underscores the theoretical and clinical significance of exploring the mechanisms linking PD and PC. It holds promise for developing innovative intervention strategies for PC prevention and treatment centered on oral health management. Based on this, the present review summarizes the epidemiological evidence, potential pathogenic mechanisms, and clinical management implications of the PD-PC association, aiming to provide a reference for relevant research and clinical practice.

## 2 Epidemiological evidence for the PD-PC association

Numerous epidemiological studies, employing diverse designs such as meta-analyses, prospective cohort studies, and case-control studies, have explored the PD-PC association across different populations and dimensions. These studies have provided substantial evidence supporting a potential link between the two conditions while initially revealing the specific characteristics of the association and its potential influencing factors (**Table 1**).

**Table 1:** Summary of core epidemiological studies on the PD-PC association.

Authors (Year/Country)	Study type	Study Population/Model	Key findings	Effect Size (HR/OR/RR and 95%CI)	Reference	Periodontal disease definition/Assessment	Key supplementary information
Maisonneuve et al. (2017/Italy)	Meta-analysis	8 relevant studies (including cohort and case-control studies)	Periodontitis and edentulism are both associated with increased PC risk; no publication bias	Periodontitis: RR = 1.74 (1.41-2.15), I²=0%, Macaskill’s test P = 0.30; Edentulism: RR = 1.54 (1.16-2.05), I²=0%, Macaskill’s test P = 0.90	(Maisonneuve et al., 2017) Ann Oncol 28(5): 985-995	Integrated heterogeneous PD assessment methods from 8 included studies: dental clinical examination (periodontal status evaluation via Russell periodontal index, plaque coverage assessment), self-report (periodontal disease history, number of remaining teeth), and healthcare administrative data (insurance claims/database coding); “periodontitis” included definitions of any periodontal disease or unacceptable dental plaque (covering >1/3 of teeth), while “edentulism” was defined as tooth loss or the lowest category of remaining teeth	The first meta-analysis systematically summarizing the PD-PC association, with cross-population generalizability
Xu et al. (2022/China)	Meta-analysis	Meta-analysis 17 observational studies (1,352,256 participants)	Oral diseases (especially PD) are associated with increased PC risk; PD contributes more than isolated tooth loss	Oral diseases: HR = 1.32 (1.13–1.54), I²=62.3%, P = 0.000; Periodontal disease: HR = 1.38 (1.12–1.71), I²=60.8%, P = 0.002; Tooth loss: HR = 1.19 (0.97–1.46), I²=67.4%, P = 0.047	(Xu et al., 2022) Pancreas 51(8): 985-994	Integrated diverse PD assessment methods from 17 included observational studies: clinical dental examinations (periodontal pocket depth, clinical attachment loss, alveolar bone resorption via radiographs, gingival bleeding index, dental plaque evaluation), self-administered questionnaires (periodontal disease history, number of remaining teeth/tooth loss), and administrative coding systems (ICD-9-CM codes 523, 523.3, 523.4 for periodontitis/gingivitis); tooth loss was defined as self-reported missing teeth or clinical assessment of edentulism	A recent meta-analysis confirmed PD as an independent risk factor for PC
Wang et al. (2024/China)	Meta-analysis	19 cohort studies (16,620,011 participants)	Periodontal diseases increase the risk of overall gastrointestinal cancers and site-specific cancers (including pancreatic cancer); severe periodontitis shows a stronger association	Overall gastrointestinal cancers: HR = 1.31 (1.16–1.49), I²=98.0%, P<0.01; Pancreatic cancer: HR = 1.35 (1.00–1.82), I²=97.0%, P<0.01; Severe periodontal diseases: HR = 1.79 (1.07–2.99), I²=0.0%, P = 0.92	(Wang Q. et al., 2024)J Dent Res 103(10): 962-972	Integrated standardized PD assessment criteria from 19 cohort studies, categorized into 5 types: clinical periodontal measurements (probing pocket depth + clinical attachment level, alveolar crestal height via radiography), medical record coding (ICD codes from administrative databases), self-reported questionnaires (periodontal disease history, gingivitis/periodontitis diagnosis), full-mouth oral examinations, and unreported assessment methods; PD subtypes included gingivitis, periodontitis, and combined gingivitis-periodontitis, with severity stratified as mild, moderate, and severe	Latest meta-analysis incorporating multi-population data
Chang et al. (2016/Taiwan, China)	Retrospective cohort study	Taiwan NHIRD database (139,805 in PD group; 75,085 in control group)	PD is associated with increased PC risk; the combined gingivitis + periodontitis subtype shows a stronger association; the association is only observed in individuals ≥65 years old	Overall: HR = 1.55 (1.02-2.33); ≥65 years old: HR = 2.17 (1.03-4.57); Gingivitis + periodontitis: HR = 1.67 (1.05-2.66)	(Chang et al., 2016) Pancreas 45(1): 134-141	Defined using ICD-9-CM diagnostic codes from Taiwan NHIRD: PD (ICD-9-CM 523), gingivitis (ICD-9-CM 523.0, 523.1), periodontitis (ICD-9-CM 523.3, 523.4), and other PD subtypes; excluded subjects with ambiguous PD status (A-code A330, which aggregates PD with non-PD oral conditions); physician-reported health claims data confirmed PD diagnosis during the exposure period (January 1, 1997-December 31, 1997)	Large-sample study in Asian population identifying age as an effect modifier; data derived from Taiwan’s National Health Insurance Research Database
Michaud et al. (2007/USA)	Prospective cohort study	U.S. male health professionals (51,529 participants; 16-year follow-up)	A history of PD is associated with increased PC risk	HR=1.64(1.19-2.26)	(Michaud et al., 2007) J Natl Cancer Inst 99(2): 171-175	Defined by self-reported questionnaire at baseline: response to the question “Have you had periodontal disease with bone loss?”The questionnaire was validated with radiographs (positive predictive value: 0.76 for dentists, 0.80 for nondentists; negative predictive value: 0.74 for dentists, 0.68 for nondentists). Biennial updates on incident tooth loss supplemented PD status during follow-up (1988-2002) as an indicator of disease severity	First validation of the PD-PC association in a large male cohort
Li et al. (2025/Taiwan, China)	Retrospective cohort study	Taiwan NHIRD database (428,814 in periodontitis group; 428,814 matched controls; follow-up 2000-2019)	No significant overall association, but significant associations are observed in subgroups of 45–64 years old, ≥65 years old, and male populations	Overall aHR=1.08 (0.96-1.22); 45–64 years old: aHR=4.43 (3.68-5.33); ≥65 years old: aHR=9.18 (7.43-11.30); Males: aHR=1.16 (1.01-1.34)	(Li et al., 2025) Li et al. BMC Oral Health (2025) 25:1856	Defined using ICD diagnostic codes from Taiwan NHIRD (ICD-9 before 2016; ICD-10 during 2016-2019) for periodontitis diagnosis; supplemented by healthcare utilization metrics: annual periodontitis medical benefit (≤USD 11, 12-27, >27) and annual outpatient (OPD) visit frequency (≤0.3, 0.4-0.7, >0.7 per year) as indicators of disease severity and treatment adequacy.	Nearly 20 years of follow-up covering 99% of Taiwan’s residents; indicating population heterogeneity in the association; lack of data on confounding factors such as lifestyle
Fan et al. (2018/USA)	Nested case-control study	U.S. CPS-II and PLCO cohorts (361 cases; 371 controls)	Colonization of P. gingivalis and A. actinomycetemcomitans is associated with increased PC risk, with a dose-response relationship; alcohol consumption may synergistically enhance the risk	P. gingivalis: OR = 1.60 (1.15-2.22); A. actinomycetemcomitans: OR = 2.20 (1.16-4.18); Former drinkers: OR = 3.03 (1.13-7.03)	(Fan et al., 2018) Gut 67(1): 120-127	No direct PD clinical diagnosis; PD-related status assessed via 16S rRNA gene sequencing of pre-diagnostic oral wash samples: targeted detection of periodontal pathogens (Porphyromonas gingivalis, A. actinomycetemcomitans, Tannerella forsythia, Prevotella intermedia) as “carriers/non-carriers”; relative abundance of PD-associated bacterial taxa (phylum to genus) quantified using the Human Oral Microbiome Database for taxonomic assignment	First direct confirmation of the prospective association between periodontal pathogens and PC via oral rinse sequencing; based on the CPS-II and the PLCO Cancer Screening Trial cohorts
Michaud et al. (2013/Multiple European countries)	Prospective cohort study (Europe)	European EPIC cohort (405 cases; 416 controls)	High antibody levels against P. gingivalis (ATCC 53978) are associated with increased PC risk; commensal bacteria may reduce the risk	OR=2.14 (1.05-4.36); High antibody levels against commensal bacteria: OR = 0.55 (0.36-0.83)	(Michaud et al., 2013) Gut 62(12): 1764-1770	No direct PD clinical diagnosis; PD-related status assessed via serological detection using ELISA-validated immunoblot array: measured plasma IgG antibody levels to 25 oral bacteria (including periodontal pathogens: P. gingivalis, A. actinomycetemcomitans, Tannerella forsythia); defined “high antibody levels” as >200 ng/ml for target pathogens, with cluster analysis used to aggregate commensal bacteria antibody profiles	First confirmation of the dose-response relationship between P. gingivalis and PC via serum antibody levels; based on the EPIC cohort
Hesami et al. (2025/Iran)	Case-control study	41 PC patients; 40 age- and gender-matched controls (without hepatobiliary diseases or cancer history)	The detection rate and quantity of P. gingivalis in saliva are higher in the case group than in the control group; the association is more pronounced in women, individuals under 50 years old, and non-diabetic patients	P. gingivalis: OR = 1.43 (1.04-1.96); Women: OR = 2.31 (0.98-5.47); Non-diabetic patients: OR = 1.67 (1.05-2.66)	(Hesami et al., 2025) Front. Cell. Infect. Microbiol. 15:1678114	No direct PD clinical diagnosis; PD-related status assessed via qPCR of unstimulated saliva samples: absolute quantification (Log10 CFU/µl) of periodontal pathogens (P. gingivalis ATCC 33277, A. actinomycetemcomitans ATCC 24523); bacterial load was validated by standard curves, melt curve analysis, and agarose gel electrophoresis for assay specificity	qPCR used to detect salivary bacterial load; first construction of a diagnostic model combining PD pathogens and miRNAs (AUC = 0.878)
Zhang Y et al. (2020/China)	Meta-analysis	Data on the association between periodontitis and PC mortality from 3 studies	Periodontitis is associated with increased PC mortality, with disease specificity (limited to PC only)	Combined HR for PC mortality=2.20 (1.44–3.37), I²=0.0%, P = 0.559	(Zhang et al., 2020) J Clin Periodontol. 2020;47:134–147	Integrated PD assessment methods from included cohort studies, categorized into two types: dental clinical examinations (periodontal probing depth, clinical attachment loss, alveolar bone resorption, and standardized periodontal evaluation) and self-reported questionnaires (periodontal disease diagnosis history, symptoms, and dental status); PD severity was classified as mild, moderate, or severe in subgroup analyses	Extremely low heterogeneity in the analysis of PC mortality association, with high result reliability; stable in high-quality and long-term follow-up studies
Heikkilä et al. (2018/Finland)	Cohort study	Finnish population (68,273 participants; 10-year follow-up)	Periodontitis is associated with increased overall cancer mortality and significantly elevated pancreatic cancer mortality; findings robust to sensitivity analyses.	Overall cancer mortality (adjusted): MRR = 1.32 (1.10-1.58); PC mortality (adjusted): MRR = 2.28 (1.31-3.98)	(Heikkilä et al., 2018) Int J Cancer 142(11): 2244-2253	Defined by two complementary criteria from Finnish national dental registers: 1) periodontitis treatment procedure codes (binary “yes/no” based on recorded clinical interventions for periodontitis); 2) clinical periodontal indices (Community Periodontal Index scores 3–4 indicating periodontal pockets >3 mm, combined with oral health metrics including decayed-missing-filled teeth index, number of teeth, and healthy/toothless sextants)	Focusing on PC mortality, first confirming the negative impact of PD on PC prognosis

PD, periodontal disease; PC, pancreatic cancer; HR, hazard ratio; OR, odds ratio; RR, relative risk; CI, confidence interval; aHR, adjusted hazard ratio; NHIRD, National Health Insurance Research Database of Taiwan, China; EPIC, European Prospective Investigation into Cancer and Nutrition cohort; CPS-II, American Cancer Society’s Cancer Prevention Study II cohort; PLCO, National Cancer Institute’s Prostate, Lung, Colorectal, and Ovarian Cancer Screening Trial cohort; ELISA, enzyme-linked immunosorbent assay; qPCR, quantitative real-time PCR. The studies in the table are ordered according to the main text. Key findings and effect sizes are extracted directly from the original articles. Key supplementary information suggests innovations and limitations to support the main text’s argumentative logic.

### 2.1 Overall association

Multiple meta-analyses suggest a potential association between PD and an increased risk of PC. An Italian meta-analysis incorporating 8 studies on periodontitis or edentulism reported a pooled relative risk (RR) of 1.74 (95% confidence interval [CI]: 1.41-2.15) for the association between periodontitis and PC, and a pooled RR of 1.54 (95% CI: 1.16-2.05) for edentulism and PC, indicating that patients with PD may have an elevated risk of developing PC (Maisonneuve et al., 2017). Two large-scale Chinese meta-analyses further validated this potential link: one including 17 observational studies found a link between oral diseases and PC (hazard ratio [HR]=1.32, 95% CI: 1.13-1.54), with subgroup analysis showing that the risk of PC in PD patients (HR = 1.38, 95% CI: 1.12-1.71) was higher than that in patients with tooth loss (HR = 1.19, 95% CI: 0.97-1.46). This suggests that PD itself may be a key driver of increased PC risk, while tooth loss is merely an indirect indicator of association (Xu et al., 2022). A 2024 meta-analysis integrating 19 cohort studies identified a modest association between PD and PC (pooled HR = 1.35, 95% CI: 1.00-1.82, P = 0.05). Notably, the 95% confidence interval includes 1.00, indicating a marginal association at the threshold of statistical significance. However, subgroup analysis revealed that severe PD was associated with a more pronounced risk elevation (pooled HR = 1.79, 95% CI: 1.07-2.99), suggesting a potential dose-response relationship between PD severity and PC risk (Wang Q. et al., 2024). Cohort study results also support this overall association trend. A retrospective cohort study using Taiwan’s National Health Insurance Research Database (NHIRD) showed that having any PD (gingivitis, periodontitis, and other PDs) was associated with an increased risk of PC (HR = 1.55, 95% CI: 1.02-2.33, P = 0.04) (Chang et al., 2016). A 16-year prospective cohort study by Michaud et al. involving over 50,000 male health professionals also found that individuals with a history of PD may have an elevated risk of PC, with a multivariate-adjusted RR of 1.64 (Michaud et al., 2007). However, a 2025 retrospective cohort study using Taiwan’s NHIRD database reported a non-significant overall association (adjusted HR = 1.08, 95% CI: 0.96-1.22) (Li et al., 2025), which aligns with the weak statistical significance observed in the 2024 meta-analysis and may reflect inherent heterogeneity in the epidemiological evidence.

The inconsistent overall associations in these two key recent studies may stem from three potential factors. First, PD definition and assessment likely varied substantially: the 2024 meta-analysis integrated standardized criteria including clinical measurements (probing pocket depth, clinical attachment level), medical record coding, and self-reported data, with explicit stratification by PD severity (mild/moderate/severe) and subtype (gingivitis/periodontitis) (Wang Q. et al., 2024); the 2025 Taiwanese cohort study relied solely on ICD-9/ICD-10 diagnostic codes supplemented by healthcare utilization metrics (annual medical benefit, outpatient visit frequency) without direct clinical periodontal evaluations (e.g., pocket depth measurement, attachment loss assessment) (Li et al., 2025), which may introduce misclassification of PD severity and subtype. Second, follow-up design and population stratification may have contributed to differences: the 2025 study conducted a nearly 20-year follow-up of a nationwide Taiwanese cohort but focused primarily on unstratified overall population analysis (Li et al., 2025), while the 2024 multi-population meta-analysis emphasized subgroup stratification by PD characteristics and population factors (Wang Q. et al., 2024); notably, both studies revealed significant associations in specific subgroups (e.g., middle-aged/elderly individuals, males) despite weak overall links, indicating the PD-PC association is likely population-specific rather than universal. Third, confounding factor control may have differed in completeness: the 2025 cohort study adjusted for demographic and socioeconomic factors (age, gender, income, Charlson Comorbidity Index) but lacked data on critical lifestyle confounders (smoking, alcohol consumption, diet) that are known to impact both PD and PC risk (Li et al., 2025), while the 2024 meta-analysis incorporated multi-study adjusted data with more comprehensive control of lifestyle and clinical confounders (Wang Q. et al., 2024), which may partially explain the slight statistical significance in the latter’s overall effect size. Collectively, current epidemiological evidence points to a potential moderate, population-specific PD-PC association: the link is statistically significant in stratified analyses (e.g., moderate-to-severe PD, high-risk populations) but weak or non-significant in unstratified overall analyses, with no definitive evidence of a strong universal causal relationship.

### 2.2 Specific associations with PD subtypes and related pathogens

Existing research has shown specific characteristics associated with PD subtypes and related pathogens. Subgroup analysis suggests that gingivitis or periodontitis is associated with an increased risk of pancreatic cancer, even after adjusting for sex, age, and other covariates (HR = 1.67; 95% CI: 1.05–2.66, P = 0.03), while no significant association was observed between other types of PD and PC risk (HR = 1.42, 95% CI: 0.87-2.30, P = 0.16) (Chang et al., 2016). At the pathogen level, a nested case-control study in the United States suggested that the carriage status of core pathogenic bacteria in periodontitis may be associated with the subsequent risk of PC. The adjusted odds ratio (OR) for PC risk was 1.60 (95% CI: 1.15-2.22) among individuals carrying P. gingivalis, with a dose-response relationship suggesting that higher carriage levels may be associated with increased risk. For individuals carrying Aggregatibacter actinomycetemcomitans (A. actinomycetemcomitans), the adjusted OR for PC risk was 2.20 (95% CI: 1.16-4.18) (Fan et al., 2018). Notably, this study ruled out reverse causality, confirming that carriage of periodontitis-related pathogens precedes the development of PC and supporting the causal inference direction of “periodontitis-related exposure → increased PC risk” (Fan et al., 2018). A large-scale prospective cohort study based on the European Prospective Investigation into Cancer and Nutrition (EPIC) cohort showed that individuals with high antibody levels (>200 ng/ml) against P. gingivalis ATCC 53978 had a 2.14-fold higher risk of PC compared to those with low antibody levels (≤200 ng/ml, OR = 2.14, 95% CI: 1.05-4.36), suggesting that the pathogenic characteristics of periodontal pathogens (such as capsular antigens) may be key to the risk association. Meanwhile, the study found that individuals with consistently high antibody levels against commensal oral bacteria had a 45% reduced risk of PC (OR = 0.55, 95% CI: 0.36-0.83), indicating that commensal bacteria may indirectly reduce PC risk by maintaining oral microbiota balance (Michaud et al., 2013). An Iranian case-control study using quantitative real-time PCR found that the detection rate (61%) and the quantity of P. gingivalis in the saliva of PC patients were higher than those in the control group (22.5%, p=0.034). The association between P. gingivalis and PC risk was more pronounced in women (OR = 2.31), individuals under 50 years of age (OR = 1.67), and non-diabetic patients (OR = 1.67). A. actinomycetemcomitans was only associated with increased PC risk in the subgroup of diabetic patients (OR = 3.66, 95% CI: 0.47-6.68) (Hesami et al., 2025).

### 2.3 Population stratification and effect modification

Population stratification and effect modification analyses suggest that the PD-PC association may exhibit population specificity, with factors such as age and alcohol exposure potentially serving as essential effect modifiers. This characteristic is crucial for clarifying the association’s applicable scope. Age-related stratified studies show that the PD-PC association is mainly concentrated in middle-aged and elderly populations: the Taiwan NHIRD cohort study indicated that the association was only statistically significant in individuals aged ≥65 years (HR = 2.17, 95% CI: 1.03-4.57, P = 0.04), suggesting that age may be an effect modifier of this association (heterogeneity test P = 0.03) (Chang et al., 2016). Subgroup analysis of another Taiwanese cohort study also validated the association in middle-aged and elderly populations, finding that periodontitis was associated with PC risk in individuals aged 45–64 years and ≥65 years (aHR=4.43 and 9.18, respectively) (Li et al., 2025). As a known risk factor for PC, the synergistic effect of alcohol exposure with PD is worthy of attention. Stratified analysis by alcohol consumption status showed that the association between A. actinomycetemcomitans and PC was stronger in former drinkers (OR = 3.03, 95% CI: 1.13-7.03, p=0.0097), suggesting that alcohol may synergistically influence PC risk with PD pathogens (Fan et al., 2018).

### 2.4 Association with PC mortality

The PD-PC association mortality is an essential dimension for evaluating the clinical significance of their relationship. Existing studies suggest a robust positive correlation between the two, with disease specificity. A Chinese meta-analysis summarizing data from 3 cohort studies showed an association between periodontitis and PC mortality (pooled HR = 2.20, 95% CI: 1.44-3.37), with extremely low heterogeneity (I²=0.0%, p=0.559), indicating good consistency across studies. Subgroup analysis further confirmed that this association was stable across high-quality, long-term follow-up, and prospective cohort studies, and was limited to PC, without a significant association with other gastrointestinal cancers, highlighting the disease specificity of the association (Zhang et al., 2020). A Finnish cohort study also validated this association. After adjusting for multiple factors such as age, gender, and socioeconomic status, PD patients had a 128% higher risk of PC-related death compared to those without PD (adjusted MRR = 2.28, 95% CI: 1.31-3.98), and sensitivity analysis further confirmed the robustness of this association (Heikkilä et al., 2018). These findings suggest that the prevention and treatment of PD may have a specific public health value in reducing PC mortality risk.

## 3 Potential mechanisms by which PD influences PC

The potential pathogenic effect of PD on PC may not be mediated through a single pathway but may act synergistically via a multi-layered network of “direct effects - mediating effects - subsequent impacts”. It may involve the potential invasion of periodontal pathogens, abnormal activation of inflammatory and immune signals, superimposed effects of flora and metabolic disorders, and potential induction of chemoresistance, ultimately promoting the occurrence, development, and poor prognosis of PC.

### 3.1 Potential colonization of periodontal pathogens and induction of precancerous lesions

**Figure 1:**
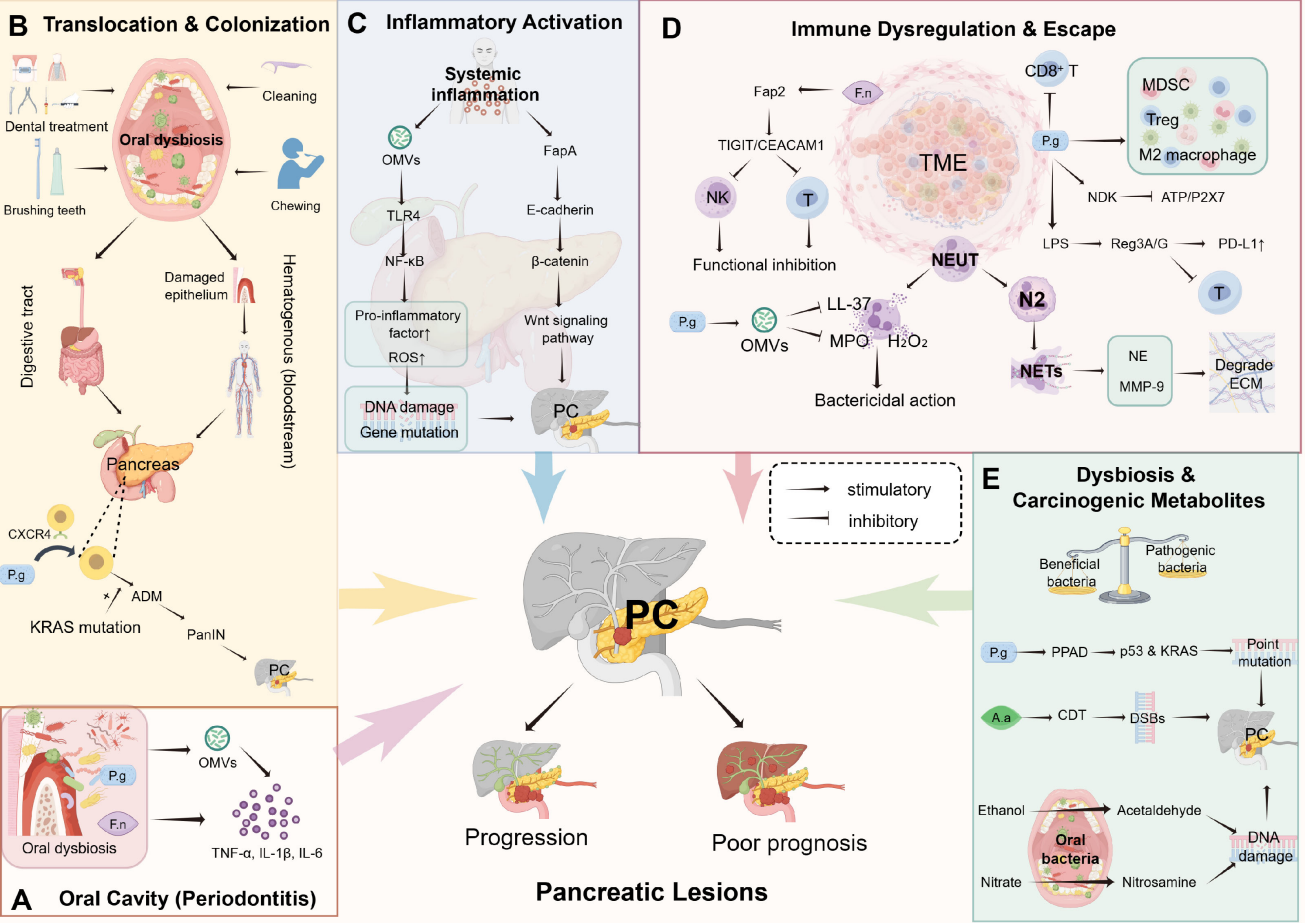
Schematic of potential mechanisms by which PD may promote PC initiation and progression. **(A)** PD-induced oral dysbiosis enriches P. gingivalis and F. nucleatum, whose cells and OMVs spread systemically. In the pancreas, four interconnected oncogenic pathways may be activated: **(B)** Translocation and colonization: Periodontal pathogens may penetrate damaged gingival epithelium, disseminate via blood or digestive tract, and colonize the pancreas; (P) gingivalis may synergize with mutant KRAS to drive ADM and PanIN. **(C)** Inflammatory activation: TLR4/NF-κB and Wnt/β-catenin signaling may be stimulated, potentially causing DNA damage and oncogene upregulation. **(D)** Immune dysregulation and escape: N2 neutrophil polarization, suppressed NK/CD8^+^ T cells, and immunosuppressive cell infiltration may remodel the TME. **(E)** Dysbiosis and carcinogenic metabolites: Bacterial enzymes (PPAD, CDT) and metabolites (acetaldehyde, nitrosamines) induce gene mutations. Together, these pathways may drive pancreatic carcinogenesis. PD, periodontal disease; PC, pancreatic cancer; P.g, Porphyromonas gingivalis; F.n, Fusobacterium nucleatum; OMVs, outer membrane vesicles; KRAS, Kirsten rat sarcoma viral oncogene homolog; ADM, acinar-to-ductal metaplasia; PanIN, pancreatic intraepithelial neoplasia; TLR4, Toll-like receptor 4; NF-κB, nuclear factor-κB; NK, natural killer cells; CD8^+^ T, CD8-positive T lymphocytes; TME, tumor microenvironment; PPAD, peptidylarginine deiminase; CDT, cytolethal distending toxin.

Periodontal pathogens can breach local oral barriers, migrate to pancreatic tissue via multiple pathways, and establish stable colonization, thereby potentially initiating or accelerating the carcinogenic process (**Figure 1**) (Kurtzman et al., 2022). Among these, Porphyromonas gingivalis (P. gingivalis) and Fusobacterium nucleatum (F. nucleatum) are the two most extensively studied bacteria for their pro-inflammatory and carcinogenic effects (Pignatelli et al., 2023). When oral flora is imbalanced, core periodontal pathogens can penetrate the damaged gingival epithelium into the systemic circulation during chewing, toothbrushing, periodontal inflammation, or dental treatment, or spread through the digestive tract after swallowing, ultimately reaching the pancreas and potentially completing colonization (Ahn et al., 2012b; Fan et al., 2018; Pignatelli et al., 2023). This “oral-pancreas” migration pathway has been initially verified through 16S rRNA sequencing and animal experiments (Tan et al., 2022; Márquez-Arrico et al., 2024). Further animal experiments have shown that, in the pancreatic microenvironment, P. gingivalis can intracellularly colonize pancreatic cells by binding to CX-chemokine receptor 4 (CXCR4) on their surfaces. It enhances the survival of tumor cells under harsh conditions, such as hypoxia and nutrient deficiency, by upregulating the expression of the glutamine transporter SLC1A5 and non-classical metabolic enzymes (GOT1, GOT2, ME1), while potentially reducing reactive oxygen species (ROS)-mediated cell death. Notably, this protective effect can be reversed by cell-penetrating antibiotics (e.g., moxifloxacin), confirming dependence on intracellular bacterial survival (Saba et al., 2024). Critically, the hypoxic microenvironment of pancreatic tumors may further enhance the intracellular survival of P. gingivalis and the proliferation of PDAC, providing an ideal niche for its survival and forming a potential vicious cycle of “bacterial colonization - microenvironmental adaptation - tumor proliferation” (Gnanasekaran et al., 2020).

Long-term exposure to periodontal pathogens may directly induce precancerous lesions in pancreatic tissue. Animal experiments have shown that after 12 weeks of oral P. gingivalis intervention in wild-type mice, pancreatic tissue exhibits acinar-to-ductal metaplasia (ADM), a key early step in pancreatic carcinogenesis characterized by high expression of ADM markers such as SOX9 and CK19, and increased mucin secretion (Saba et al., 2024). More importantly, the study found that after treatment with gentamicin + metronidazole, viable bacteria remained only in KRASG12D-positive cells, suggesting that the bacteria may synergize with the core oncogenic mutation KRASG12D in PC. Additionally, intracellular bacteria may reduce the ROS elevation induced by KRASG12D, thereby evading oncogene-mediated cellular senescence and ultimately accelerating the progression of pancreatic intraepithelial neoplasia (PanIN) to PDAC (Saba et al., 2024). In infected mice, most lesions progressed to high-grade PanIN or typical PDAC. In contrast, only low-grade PanIN was observed in uninfected mice, suggesting that the synergistic effect between the two may be an essential driver of carcinogenesis (Saba et al., 2024). Furthermore, long-term exposure to P. gingivalis may reshape the composition of the pancreatic microbiome, increasing the relative abundance of potential pro-carcinogenic flora such as Mycoplasmataceae and Helicobacteraceae, and further optimizing the carcinogenic microecological environment (Saba et al., 2024). In humans, studies have shown that P. gingivalis is enriched in the intratumoral flora of human PC tissues and can also be detected in saliva samples, normal tissues, and malignant tissues of PC patients, further supporting the relevance of the colonization pathway of periodontal pathogens from the oral cavity to the pancreas (Ungureanu et al., 2023).

### 3.2 Potential activation of inflammation-mediated carcinogenic pathways

Inflammation plays a key role in creating an environment conducive to cancer development, with up to 20% of cancers associated with chronic infections (Singh et al., 2019). Persistent inflammation caused by insufficient regulation of immune system responses may disrupt the cellular environment (sustained disturbance of the cellular microenvironment), leading to changes in cancer-related genes and alterations in key proteins that control the cell cycle, DNA repair, and apoptosis (Eiró and Vizoso, 2012). The PC itself is closely associated with a chronic inflammatory state; thus, chronic inflammation may serve as an essential mediator linking PD and PC. PD can construct a pro-carcinogenic microenvironment and potentially activate key carcinogenic pathways through local inflammatory spread and systemic inflammatory responses (Maisonneuve et al., 2017; Xu et al., 2022).

In periodontitis, outer membrane vesicles (OMVs) secreted by P. gingivalis and other important PD pathogens can disrupt host structures, induce host immune responses, and promote the progression of periodontitis (Zhang L. et al., 2024). Studies have shown that OMVs secreted by PD pathogens can activate Toll-like receptor 4 (TLR4) and its downstream targets ERK, CREB, and Nuclear Factor-κB (NF-κB), thereby promoting the production of pro-inflammatory cytokines (Engevik et al., 2021). In the oral cavity, periodontal pathogens and their secreted OMVs can induce gingival tissues to produce pro-inflammatory cytokines such as TNF-α, IL-1β, and IL-6 (**Figure 1**) (Chung et al., 2019; Chattopadhyay et al., 2023). These inflammatory mediators spread systemically through the bloodstream, leading to sustained elevation of systemic inflammatory markers (C-reactive protein, IL-6, TNF-α) (Meyer et al., 2008; El Ayachi et al., 2025). OMVs secreted by Gram-negative bacteria such as P. gingivalis and A. actinomycetemcomitans typically contain lipopolysaccharide (LPS), DNA, adhesins (Fap), and various enzymes (Ellis and Kuehn, 2010). In pancreatic tissue, TLR4 on pancreatic cells can recognize LPS, triggering the release of inflammatory cytokines and the production of ROS through NF-κB signaling, which, in turn, leads to DNA damage and gene mutations, accelerating the occurrence of PC (Baima et al., 2023). Additionally, TLR activation is a key driver of PC development; activating this pathway can induce chronic pancreas inflammation, promote pancreatic stellate cell activation, and further drive the malignant transformation of pancreatic epithelial cells and the formation of the tumor microenvironment (TME, Zambirinis et al., 2015). Beyond LPS, studies have shown that FapA of F. nucleatum can bind to E-cadherin on host epithelial cells, activating Wnt signaling by promoting nuclear accumulation of β-catenin. Specifically, β-catenin-regulated transcription factors interact with Lymphoid Enhancer Factor (LEF)/T-Cell Factor (TCF) to activate Wnt target genes such as c-myc and cyclin D1, thereby initiating PC development (**Figure 1**) (Chattopadhyay et al., 2023).

Furthermore, inflammation-mediated epigenetic regulation may further exacerbate the carcinogenic process. Kanli et al. conducted a network analysis focusing on miRNA regulatory pathways shared between periodontitis and prostate cancer, and their findings have shown that the chronic pro-inflammatory microenvironment induced by periodontitis can trigger abnormal expression of specific microRNAs (miRNAs). These miRNAs can be transported to pancreatic tissue via exosomes to modulate gene expression. Notably, this research did not directly validate the functions of these miRNAs in pancreatic cancer or the crosstalk between PD and PC (Kanli et al., 2025). Further studies have shown that there are 20 differentially expressed miRNAs shared between PD and PC, among which hsa-miR-155 is a core node molecule that may target and regulate key genes such as SOD2 (epithelial-mesenchymal transition), TXNIP (ROS-NLRP3 inflammation), and CCND1 (cell cycle), as well as transcription factors such as AKT1 (cell survival), BCL6 (apoptosis resistance), and BRCA1 (DNA repair (Sunnetci-Akkoyunlu et al., 2025). However, direct functional validation of these miRNAs in PD-PC-associated models remains lacking; their potential roles in modulating PC cell proliferation, oxidative stress, or chemoresistance are inferred rather than experimentally confirmed.

### 3.3 Potential risks of immune dysregulation and tumor immune escape

Periodontal pathogens may reshape the TME and may activate immune escape pathways, potentially impairing the body’s anti-tumor immune capacity and may provide potential support for PC progression (**Figure 1**).

Pathogens such as P. gingivalis and F. nucleatum can regulate immune cell function through multiple mechanisms. On one hand, these bacteria can significantly increase the number of neutrophils. Studies have shown that OMVs secreted by periodontal pathogens not only induce inflammatory responses but also serve as immune-evasion tools, enabling bacteria to evade immune attacks. The antigenic determinants retained on their surfaces are highly immunogenic and can act as decoys, binding antibodies and complement and depleting them, thereby protecting bacteria from immune attack during infection (Nakao et al., 2011). Additionally, OMVs can serve as regulators of gingival immune responses, modulating monocyte immune activity and inducing immune tolerance. If the host is subsequently re-stimulated by P. gingivalis, monocytes pre-stimulated by its OMVs will be unable to secrete tumor necrosis factor (TNF) in response (Zhang et al., 2025). Thus, sustained exposure to P. gingivalis OMVs in periodontal diseases may induce selective TNF deficiency, thereby impairing the host’s ability to recognize microorganisms (Waller et al., 2016). Neutrophils are the most abundant type of white blood cell recruited to the gingival sulcus in response to periodontal pathogens, accounting for over 95% of the total. They are the core cells of periodontal immune defense (Hajishengallis et al., 2015). Studies have shown that OMVs secreted by P. gingivalis can specifically bind to the surface of neutrophils and induce their degranulation independently of gingipains and plasma, thereby releasing antibacterial substances such as myeloperoxidase (MPO), antimicrobial peptide (LL-37), and hydrogen peroxide (H_2_O_2_). Subsequently, OMVs mediate the citrullination of arginine at position 569 of MPO via the peptidylarginine deiminase (PAD) carried by OMVs, leading to MPO inactivation and degradation and thereby blocking the bactericidal effect of MPO-H_2_O_2_; in addition, OMVs can degrade LL-37. Through these two parallel pathways, OMVs can disrupt neutrophil antibacterial mechanisms, ultimately enabling immune escape (du Teil Espina et al., 2022).

Animal model studies have shown that exposure to P. gingivalis can accelerate tumor growth in orthotopic and subcutaneous PC mouse models, and the cancerous pancreatic tissue exhibits a pro-inflammatory TME dominated by neutrophils (Tan et al., 2022). The bacterium recruits neutrophil aggregation and induces their polarization towards the pro-tumor N2 subgroup (high expression of S100a8 and S100a9 proteins) by promoting the secretion of neutrophil chemokines (Cxcl1, Cxcl2, Cxcr2) and neutrophil elastase (NE). Simultaneously, it downregulates genes involved in Gram-negative bacterial defense and anti-tumor functions (such as lymphocyte chemotaxis and phagocytosis), impairing the body’s anti-tumor immune capacity and promoting pancreatic tumor progression (Tan et al., 2022). In the cancerous environment, N2 neutrophils, as an immunosuppressive cell subgroup, secrete NE, a neutrophil extracellular trap (NET)-related protease that can promote tumor invasion by degrading the extracellular matrix (ECM, Wang et al., 2018; Márquez-Arrico et al., 2024). Additionally, studies have found that gingipains secreted by P. gingivalis can stimulate protease-activated receptor 2 (PAR2), activating the PAR2/NF-κB signaling pathway; after invading host cells, it can also activate the erk1/2-Ets1 and p38/HSP27 pathways. These three pathways collectively induce neutrophils to secrete matrix metalloproteinase precursor (proMMP-9). Subsequently, proMMP-9 is released extracellularly via the PAR2 pathway, and activated MMP-9 can also degrade various ECM components, thereby promoting PDAC invasion and metastasis (Sun et al., 2019).

On the other hand, these bacteria may inhibit the anti-tumor activity of immune cells such as T cells and natural killer (NK) cells (Baima et al., 2024). F. nucleatum may bind to TIGIT (a receptor with immunoglobulin and ITIM domains expressed on specific T cells and NK cells) and CEACAM1 receptors through its Fap2 protein, inhibiting the anti-tumor functions of NK cells and T cells and inducing lymphocyte apoptosis, thereby impairing the ability of these immune cells to kill tumor cells (Garrett, 2015; Alkharaan et al., 2020; Pignatelli et al., 2023). Furthermore, tumor invasion by P. gingivalis may reduce the number of CD8^+^ T cells, further weakening the anti-tumor immune response (Márquez-Arrico et al., 2024).

The recruitment of immunosuppressive cell populations may further exacerbate immune dysregulation. P. gingivalis may recruit immunosuppressive cells such as myeloid-derived suppressor cells (MDSCs), regulatory T cells (Treg), and M2-type macrophages by activating the miR-21/PTEN signaling axis, forming an immunosuppressive TME dominated by these cells (Zou et al., 2025). Meanwhile, functional molecules secreted by the bacterium can directly regulate immune responses: its secreted nucleoside diphosphate kinase (NDK) can consume ATP, blocking ATP/P2X7 signaling on cancer cells and immune cells, inhibiting P2X7 receptor-mediated cancer cell apoptosis, and promoting cancer cell survival (Deng et al., 2025). Additionally, P. gingivalis can induce the overexpression of miR-203, inhibiting the expression of suppressor of cytokine signaling 3 (SOCS3), and driving the activation of anti-apoptotic genes through STAT3, further enhancing the immunosuppressive effect (Chattopadhyay et al., 2023).

Furthermore, studies have shown that LPS from P. gingivalis can stimulate the upregulated expression of Reg3A/G, and these proteins may play key roles in PD-related PC, serving as another key mediator of immune dysregulation (Hiraki et al., 2020). In pancreatic tissue, Reg3A/G can be highly expressed in ADM tissue: Reg3A can promote the ductal metaplasia transformation of mouse acinar cells, while Reg3G exerts an immuno-promoting effect by inhibiting the anti-tumor effect of T cells (Li et al., 2016; Liu et al., 2017). Both can not only induce the formation of precancerous lesions but also impair dendritic cell maturation and T cell-mediated anti-tumor responses, activate the EGFR/JAK2/STAT3 signaling pathway, disrupt cell cycle progression, and enhance glycolysis, ultimately inducing the upregulation of PD-L1 and T cell dysfunction, promoting immune escape (Zou et al., 2025). Notably, Reg3A can be upregulated in tumor tissue from PC patients with gemcitabine resistance, and its deletion can enhance cancer cells’ sensitivity to gemcitabine. Thus, Reg3A can also serve as a potential biomarker of gemcitabine (GEM) resistance in PC, offering a new approach to therapeutic intervention and patient stratification (Liu et al., 2025). Additionally, P. gingivalis and F. nucleatum may further weaken the body’s ability to eliminate cancerous cells by reducing the level of p53 tumor suppressor protein and enhancing anti-apoptotic characteristics, creating favorable conditions for tumor progression (Teles et al., 2020; Baima et al., 2024).

### 3.4 Potential carcinogenic effects of flora imbalance and metabolites

Oral flora imbalance is a core feature of PD and may also represent a key mediator of PC (Olsen and Yilmaz, 2019). Under conditions of oral flora homeostasis, the microbial community possesses a sophisticated immune communication system that enables mutual collaboration with the host (Hu et al., 2025). To maintain oral flora homeostasis, oral bacteria and the host may participate in protective immune responses together. For example, F. nucleatum can induce the production of antimicrobial peptides or cytokines by activating TLRs on oral epithelial cells, thereby maintaining oral homeostasis and health (Ghosh et al., 2018). However, oral bacteria may also evade or disrupt host immune responses, leading to an imbalance in the flora. Oral flora imbalance may exert potential carcinogenic effects through excessive proliferation of pathogenic bacteria, reduced levels of beneficial bacteria, and abnormal accumulation of metabolites (Maisonneuve et al., 2017; Mascitti et al., 2019; Ungureanu et al., 2023; Fanijavadi and Jensen, 2025). Moreover, an imbalance in the flora can inhibit the body’s anti-tumor immunity against PC, thereby accelerating cancer progression (Ikenaga et al., 2024).

In the state of PD, the abnormal proliferation of pathogenic bacteria, such as P. gingivalis, may disrupt oral flora balance by competitively excluding beneficial bacteria, such as Capnocytophaga ochracea and Veillonella, and by promoting the colonization of other pathogenic bacteria (Michaud et al., 2013; Maisonneuve et al., 2017). Even in individuals without obvious PD symptoms, elevated antibody levels against these pathogenic bacteria may be associated with an increased risk of PC (Maisonneuve et al., 2017). Additionally, studies have shown that even extremely low levels of P. gingivalis (less than 0.01% of the total bacterial population) can induce flora imbalance by affecting the function of the host complement system (Hajishengallis et al., 2011). Animal experiments have further shown that P. gingivalis can alter the composition of the pancreatic microbiome, increasing the relative abundance of PDAC-related bacterial families (Mycoplasmataceae, Helicobacteraceae, Prevotellaceae) in the pancreas of wild-type and iKC mice (PDAC genetic engineering models), forming a microecological environment conducive to carcinogenesis (Saba et al., 2024). Furthermore, oral microorganisms may provide immune protection for themselves or other flora by inhibiting the host immune system and disrupting microbial homeostasis (Hu et al., 2025). For example, F. nucleatum may act as a connecting bridge between early colonizing bacteria, A. actinomycetemcomitans, and late colonizing bacteria, P. gingivalis, thereby jointly promoting the development of PC (Chattopadhyay et al., 2023).

In addition to causing flora imbalance, periodontal pathogens and their metabolites may directly or indirectly activate carcinogenic pathways, promoting the occurrence and development of PC (**Figure 1**). On the one hand, specific enzymes secreted by oral bacteria may induce gene damage. For example, PPAD secreted by P. gingivalis and other bacteria can degrade arginine, potentially leading to point mutations in the p53 and KRAS genes, among which p53 arginine mutations are significantly more common in PC patients (Öğrendik, 2017). In PDAC, KRAS is the most frequently mutated oncogene; KRAS mutations can drive cancer cell proliferation, metabolic reprogramming, immune escape, and therapeutic resistance, and are core driving factors of PDAC (Zhang et al., 2023). Specifically, KRAS mutations can inhibit the number and function of T cells by regulating the tumor microenvironment, thereby promoting immune escape (Zhang et al., 2023). In advanced PDAC, to cope with higher intracellular oxidative stress, KRAS mutations can also upregulate NRF2 expression, thereby inducing chemoresistance (Mukhopadhyay et al., 2020). Cytolethal distending toxin (CDT) produced by A. actinomycetemcomitans can induce DNA double-strand breaks (DSBs), triggering genomic instability (Faïs et al., 2016; Teshima et al., 2018; Chattopadhyay et al., 2023), and its secreted leukotoxin A (LtxA) can induce apoptosis by binding to β2 integrin LFA-1 (lymphocyte function-associated antigen-1) on antigen-presenting cells, participating in immune escape (Vega et al., 2019).

On the other hand, oral bacteria may generate or activate carcinogens through metabolic activities. For example, oral streptococci can convert ethanol into the class I carcinogen acetaldehyde, which acts on pancreatic tissue after gastrointestinal absorption, inducing DNA damage (Maisonneuve et al., 2017). Additionally, oral bacteria can activate N-nitroso compounds (potent carcinogens) in tobacco smoke and food; among these, oral nitrate-reducing bacteria account for nearly 80% of human nitrite exposure and can catalyze the formation of endogenous N-nitroso compounds in the stomach (Maisonneuve et al., 2017). Studies have also found that endogenous nitrosamine production is significantly higher in individuals with poor oral hygiene than in those with good oral hygiene, further amplifying the carcinogenic effect (Xu et al., 2022). Furthermore, smoking can enhance oral bacteria-mediated alcohol-related acetaldehyde production, which may be one of the reasons for the synergistic effect between alcohol and tobacco in carcinogenesis (Ahn et al., 2012a). In addition to the above metabolites, oral bacterial metabolites, such as reactive oxygen sulfides and butyrate, may also induce inflammatory responses and promote tumor formation through mechanisms including DNA alkylation, mutation, damage, and repair disorders (Teles et al., 2020).

Notably, PD-induced tooth loss may alter eating habits, thereby indirectly increasing PC susceptibility (Xu et al., 2022). Individuals with edentulism or tooth loss may reduce their intake of fruits and vegetables and increase their intake of high-calorie, high-fat foods due to chewing difficulties. Long-term malnutrition may further exacerbate PC risk by affecting intestinal nutrient absorption (Meyer et al., 2008; Xu et al., 2022).

### 3.5 Potential induction of chemoresistance and prognostic impact

**Figure 2:**
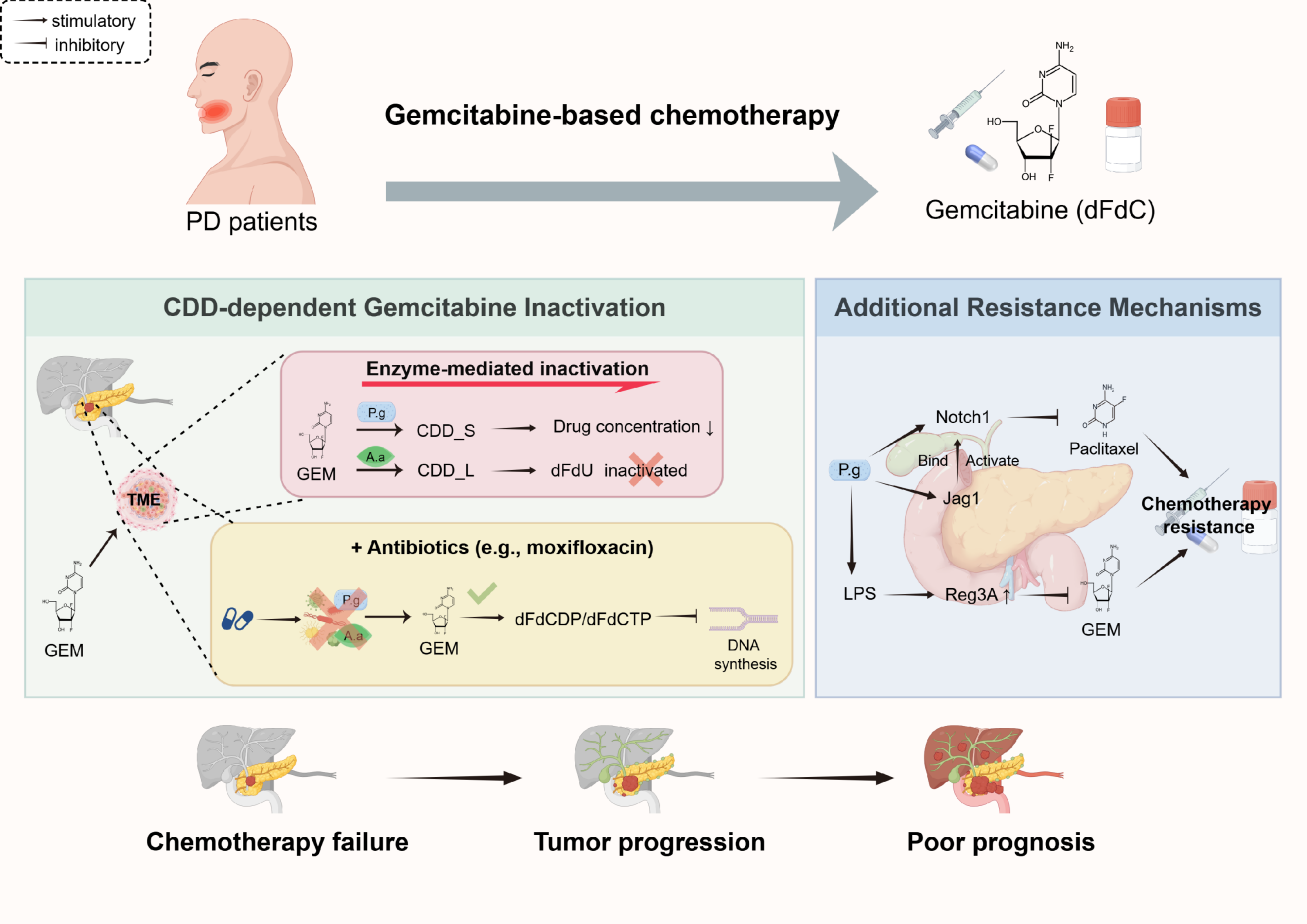
Periodontal pathogens may induce chemoresistance in PC. This schematic illustrates how periodontal pathogens in the TME may impair chemotherapeutic efficacy in PC. Key pathogens P. gingivalis and A. actinomycetemcomitans express CDD, which inactivates dFdC into inactive dFdU, potentially reducing effective drug concentration. This resistance may be reversible with antibiotic co-treatment. Additional mechanisms may include P. gingivalis-induced Notch1 pathway activation (potentially mediating paclitaxel resistance) and LPS-driven Reg3A upregulation (potentially linked to gemcitabine resistance). Collectively, these processes may lead to chemotherapy failure, tumor progression, and poor prognosis. PC, pancreatic cancer; A.a, Aggregatibacter actinomycetemcomitans; CDD, cytidine deaminase; CDD_L, long-chain cytidine deaminase; CDD_S, short-chain cytidine deaminase; dFdC, gemcitabine; dFdU, 2’,2’-difluorodeoxyuridine; GEM, gemcitabine; Jag1, Jagged1; LPS, lipopolysaccharide; Notch1, notch receptor 1; PD, periodontal disease; PDAC, pancreatic ductal adenocarcinoma; TME, tumor microenvironment.

Periodontal pathogens may not only contribute to the occurrence and progression of PC but may also induce chemoresistance via specific molecular mechanisms, thereby exacerbating poor patient prognosis (**Figure 2**). Gemcitabine (GEM) is a first-line chemotherapeutic agent for PC, and its efficacy depends on the stable presence of its active forms (the 5’-diphosphate derivative dFdCDP and the triphosphate derivative dFdCTP) generated by intracellular metabolism (Zeng et al., 2019). As a pyrimidine analog, the active metabolite of GEM, dFdCTP, exerts anti-PC effects by competitively incorporating into DNA strands to terminate their synthesis, inhibiting ribonucleotide reductase to deplete DNA replication substrates, arresting the tumor cell cycle at the S phase and G1/S phase junction, and inducing apoptosis (Beutel and Halbrook, 2023). P. gingivalis and A. actinomycetemcomitans can express the short-chain form (CDD_S) and long-chain form (CDD_L) of cytidine deaminase, respectively (Choy et al., 2018). Among these, CDD_L can completely convert GEM into inactive 2’,2’-difluorodeoxyuridine (dFdU), while CDD_S can only partially metabolize gemcitabine. However, its continuous expression in the tumor microenvironment can still significantly reduce the effective drug concentration (Xu et al., 2025). Studies by Geller et al. have confirmed that bacteria expressing CDD can directly weaken the anti-proliferative activity of GEM, and combination therapy with antibiotics can reverse this resistant phenotype, suggesting that the intracellular colonization of periodontal pathogens and CDD expression may be one of the essential reasons for the failure of clinical GEM chemotherapy (Geller et al., 2017).

In addition to GEM, periodontal pathogens may mediate paclitaxel resistance through non-CDD-dependent mechanisms. The combination of GEM and the new-generation protein-bound paclitaxel (nab-paclitaxel) is a core drug combination for the treatment of metastatic pancreatic ductal adenocarcinoma (mPDAC, Zhang D. et al., 2024). Studies have shown that high Notch1 expression is significantly associated with paclitaxel resistance (P < 0.01, Zhang et al., 2019). P. gingivalis can induce paclitaxel resistance in oral squamous cell carcinoma by activating the Notch1 signaling pathway (Woo et al., 2017). Specifically, P. gingivalis can upregulate the expression of the Notch1 receptor and its ligand Jagged1 (Jag1); simultaneously, it can induce Jag1 release, further activating Notch1 through paracrine signaling. Additionally, after the binding of Jag1 to Notch1, gingipain proteases produced by P. gingivalis can cleave the extracellular domain of Notch1, thereby promoting the activation of the Notch signaling pathway (Fitzsimonds et al., 2021). Since the Notch1 pathway is widely expressed in PC cells and closely associated with tumor recurrence and chemoresistance, Notch1 activation by P. gingivalis may represent an essential supplementary mechanism of resistance to combined chemotherapy regimens (Zhai et al., 2023).

Furthermore, 5-fluorouracil, one of the PC chemotherapeutic drugs, may exacerbate periodontal inflammation damage, and the persistent systemic inflammation induced by PD may further weaken treatment tolerance, forming a potential reciprocal detrimental cascade of “chemotherapy-induced damage - periodontal inflammation - treatment resistance” (Sunnetci-Akkoyunlu et al., 2025).

### 3.6 Evidence level rating for potential mechanistic pathways linking PD and PC

To clarify the strengths and limitations of current research into the pathogenic mechanisms linking PD and PC, we classify and rate all potential core mechanistic pathways into three tiers. The first is strong human evidence, supported by direct clinical, molecular, or epidemiological data from human PC patients with consistent results across multiple studies. The second is indirect evidence, supported by preclinical data such as animal models and *in vitro* PC cell experiments, or extrapolated from valid research in other cancer types and systemic inflammatory diseases, with only preliminary human correlative data and no direct functional validation. The third is still speculative, supported only by preliminary *in vitro* findings or single small-sample studies, with no direct validation in PC models or human samples, and the underlying mechanism is unconfirmed and controversial. A detailed rating of all pathways is presented in **Table 2**.

**Table 2:** Evidence level rating for potential mechanistic pathways linking PD and PC.

Core mechanistic pathway	Evidence level	Key supporting evidence basis	References
P. gingivalis/A. actinomycetemcomitans translocation and colonization in pancreatic tissue/cells	Strong human evidence	1. P. gingivalis detected in human PC tissue, saliva via 16S rRNA/qPCR; 2. Pre-diagnostic salivary pathogen carriage correlates with PC risk (dose-response); 3. Elevated anti-P. gingivalis IgG in PC patients; 4. P. gingivalis binds CXCR4 for pancreatic cell colonization	(Michaud et al., 2013; Fan et al., 2018; Kurtzman et al., 2022; Tan et al., 2022; Márquez-Arrico et al., 2024; Hesami et al., 2025)
PD inflammatory mediators activate TLR4/NF-κB in pancreas to induce DNA damage/oncogene expression	Strong human evidence	1. Elevated IL-6/CRP/TNF-α in PD-PC patients linked to poor prognosis; 2. TLR4/NF-κB activation in PC tissue correlates with PD severity; 3. Periodontal pathogen LPS detected in peripheral blood; 4. Pathogen OMVs activate TLR4/NF-κB to induce ROS-DNA damage	(Ellis and Kuehn, 2010; Zambirinis et al., 2015; Maisonneuve et al., 2017; Chung et al., 2019; Xu et al., 2022; Baima et al., 2023; Chattopadhyay et al., 2023)
Periodontal pathogen CDD mediates gemcitabine inactivation and GEM resistance	Strong human evidence	1. P. gingivalis/A. actinomycetemcomitans express CDD_S/CDD_L in PC tissue (correlates with GEM resistance); 2. Antibiotic combination reverses GEM resistance in clinical pilots; 3. Elevated dFdU in plasma of PD patients on GEM; 4. CDD reduces effective GEM concentration *in vitro*	(Geller et al., 2017; Choy et al., 2018; Beutel and Halbrook, 2023; Xu et al., 2025)
PD oral dysbiosis alters pancreatic microbiome and enriches pro-carcinogenic flora	Indirect evidence	1. P. gingivalis intervention reshapes pancreatic microbiome (pro-carcinogenic flora enrichment) in mice; 2. Oral dysbiosis in PD patients correlates with abnormal pancreatic microbiome; 3. Low-abundance P. gingivalis induces oral dysbiosis via the complement system; 4. Pancreatic pro-carcinogenic flora links to PC incidence	(Hajishengallis et al., 2011; Michaud et al., 2013; Maisonneuve et al., 2017; Olsen and Yilmaz, 2019; Thomas and Jobin, 2020; Saba et al., 2024)
Periodontal pathogens induce pancreatic ADM/PanIN and synergize with KRAS mutation	Indirect evidence	1. P. gingivalis induces pancreatic ADM (SOX9/CK19 upregulation) in mice; 2. Synergizes with KRASG12D to accelerate PanIN-PDAC progression in mice; 3. P. gingivalis enriched in human PanIN (correlates with lesion grade)	(Ungureanu et al., 2023; Saba et al., 2024)
Periodontal pathogens reshape PC TME, induce N2 neutrophil polarization and inhibit CD8^+^T/NK function	Indirect evidence	1. P. gingivalis induces N2 polarization and reduces CD8^+^T/NK infiltration in mouse PC models; 2. Pathogen OMVs inactivate neutrophil bactericidal system *in vitro*; 3. Gingipains induce proMMP-9 secretion to promote invasion; 4. PD severity correlates with TME immunosuppression in humans	(Sun et al., 2019; du Teil Espina et al., 2022; Tan et al., 2022; Baima et al., 2024; Márquez-Arrico et al., 2024; Zou et al., 2025)
F. nucleatum FapA/Fap2 drives PC carcinogenesis and immune escape	Indirect evidence	1. FapA binds E-cadherin, activates Wnt/β-catenin, upregulates c-myc/cyclin D1 to initiate PC; 2. Fap2 binds TIGIT/CEACAM1, inhibits NK/CD8^+^T function and induces lymphocyte apoptosis; 3. F. nucleatum detected in human PC tissue and linked to TME immunosuppression	(Garrett, 2015; Alkharaan et al., 2020; Chattopadhyay et al., 2023; Pignatelli et al., 2023)
P. gingivalis activates the Notch1 pathway to induce paclitaxel resistance	Indirect evidence	1. P. gingivalis activates Notch1 to induce paclitaxel resistance in oral cancer; 2. High Notch1 correlates with paclitaxel resistance in PC cells; 3. P. gingivalis abundance correlates with Notch1 activation in human PC tissue; 4. Notch1 may affect GEM+nab-paclitaxel efficacy	(Woo et al., 2017; Zhang et al., 2019; Fitzsimonds et al., 2021; Zhai et al., 2023)
PD-related exosomal miRNAs regulate PC cell biological behavior	Still speculative	1. 20 shared differentially expressed miRNAs identified (hsa-miR-155 as core); 2. hsa-miR-155 regulates PC cell proliferation/oxidative stress *in vitro*; 3. Elevated miRNAs in PD patients’ peripheral blood exosomes; 4. Circulating miRNAs predict PC risk 5 years in advance	(Wang C. et al., 2024; Kanli et al., 2025; Sunnetci-Akkoyunlu et al., 2025)
Periodontal pathogen metabolites/enzymes induce PC gene mutations/genomic instability	Still speculative	1. Acetaldehyde/nitrosamines induce DNA damage in pancreatic tissue; 2. PPAD induces p53/KRAS point mutations, CDT causes DNA double-strand breaks; 3. Elevated carcinogenic metabolites in PD patients’ peripheral blood; 4. Oral nitrate-reducing bacteria link to PC risk	(Faïs et al., 2016; Öğrendik, 2017; Maisonneuve et al., 2017; Teshima et al., 2018; Xu et al., 2022; Chattopadhyay et al., 2023)
P. gingivalis upregulates Reg3A/G to induce PC immune escape and chemoresistance	Still speculative	1. P. gingivalis LPS upregulates Reg3A/G and induces ADM in mouse pancreas; 2. Reg3A upregulation correlates with GEM resistance (deletion restores sensitivity) *in vitro*; 3. Reg3A/G impairs T cell function and upregulates PD-L1; 4. Elevated Reg3A in PD-PC patients linked to poor prognosis	(Li et al., 2016; Liu et al., 2017; Hiraki et al., 2020; Liu et al., 2025; Zou et al., 2025)

PD, periodontal disease; PC, pancreatic cancer; P. gingivalis, Porphyromonas gingivalis; A. actinomycetemcomitans, Aggregatibacter actinomycetemcomitans; CXCR4, C-X-C chemokine receptor type 4; IL-6, interleukin-6; CRP, C-reactive protein; TNF-α, tumor necrosis factor-α; TLR4, toll-like receptor 4; NF-κB, nuclear factor-κB; LPS, lipopolysaccharide; OMVs, outer membrane vesicles; ROS, reactive oxygen species; CDD, cytidine deaminase; CDD_S, short-chain cytidine deaminase; CDD_L, long-chain cytidine deaminase; GEM, gemcitabine; dFdU, 2’,2’-difluorodeoxyuridine; ADM, acinar-to-ductal metaplasia; PanIN, pancreatic intraepithelial neoplasia; PDAC, pancreatic ductal adenocarcinoma; TME, tumor microenvironment; NK, natural killer cell; MMP-9, matrix metalloproteinase-9; F. nucleatum, Fusobacterium nucleatum; TIGIT, T cell immunoglobulin and ITIM domain; PPAD, peptidylarginine deiminase; CDT, cytolethal distending toxin.

## 4 Clinical management implications

**Figure 3:**
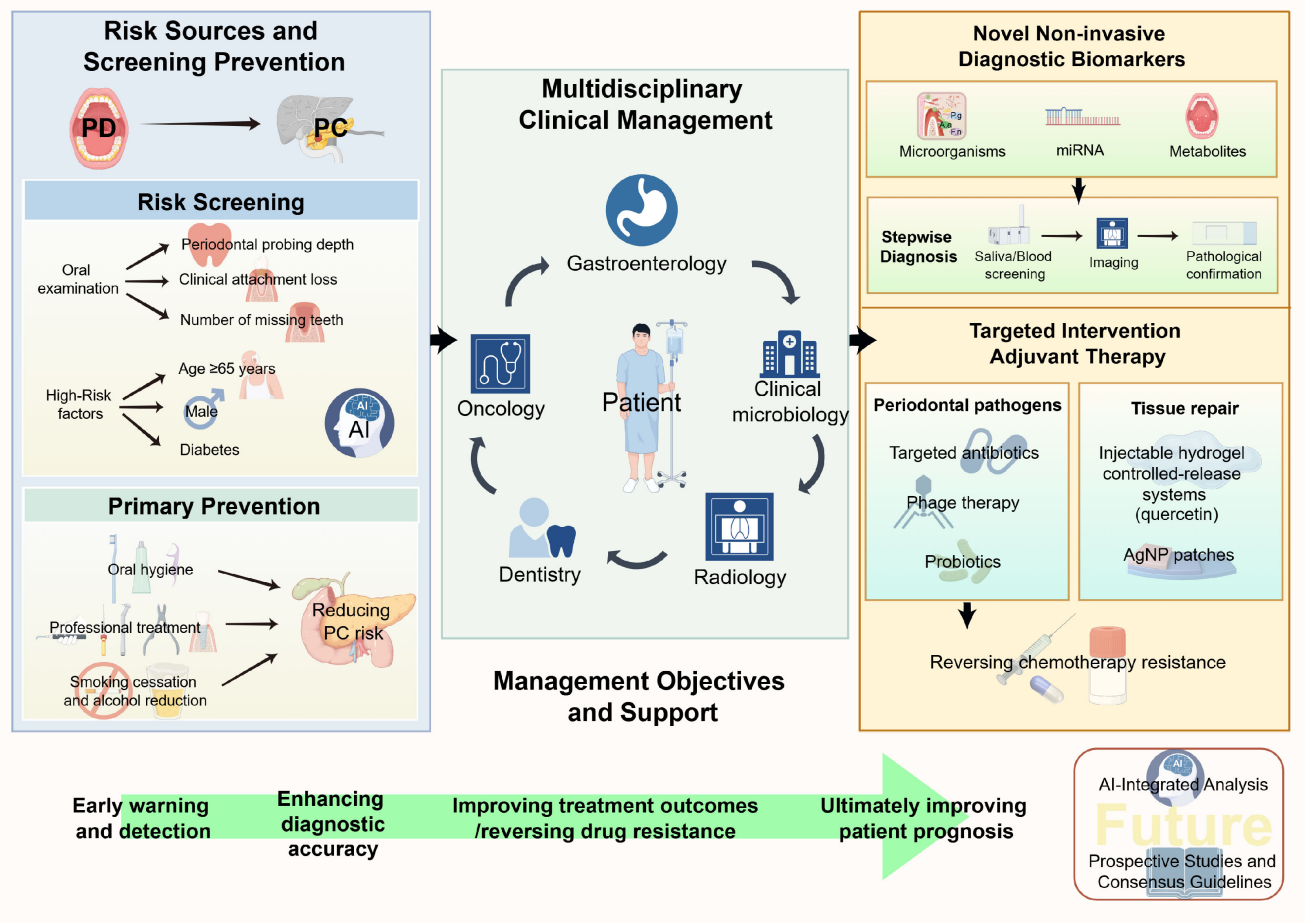
Translational Clinical Management Framework for PD and PC Association. This diagram illustrates a cross-organ collaborative clinical management system linking oral and pancreatic health based on epidemiological and mechanistic associations that suggest a potential link. Centered on MDT collaboration, it integrates three implementation pathways: risk screening and prevention, diagnostic biomarker optimization, and therapeutic strategy innovation. Through coordinated operations among “Dentistry-Oncology-Gastroenterology-Imaging-Microbiology,” this framework may achieve early warning, precision diagnosis and treatment, and reversal of drug resistance, potentially improving the prognosis for pancreatic cancer patients. PD, periodontal disease; PC, pancreatic cancer; AI, artificial intelligence; miRNA, microRNA; AgNP, silver nanoparticles.

Based on the epidemiological association and multi-dimensional pathogenic mechanisms between PD and PC, relevant research findings provide a novel perspective for the clinical prevention and control of PC. A cross-organ collaborative management system for “oral-pancreas” can be established through risk screening, diagnostic optimization, treatment strategy adjustment, and multidisciplinary collaboration (MDT), providing practical support for improving PC prognosis (**Figure 3**).

### 4.1 Optimization of risk screening and prevention

PD can serve as a potential risk marker for PC and be incorporated into a multi-factor risk screening system to provide targets for early intervention. Epidemiological studies have shown that individuals with moderate to severe periodontitis or tooth loss ≥5 have a significantly increased risk of PC (Gerlovin et al., 2019; Xu et al., 2022; Wang Q. et al., 2024). This association is more pronounced in high-risk groups, such as individuals aged ≥65 years, males, or those with comorbid diabetes (Chang et al., 2016; Gerlovin et al., 2019; Li et al., 2025). Therefore, clinical assessment of periodontal health, including indicators such as periodontitis diagnosis, number of missing teeth, periodontal probing depth, and clinical attachment loss, can be included in the routine screening process for high-risk PC populations (El Ayachi et al., 2025). For high-risk individuals with concurrent PD, it is recommended to strengthen targeted PC screening, such as abdominal ultrasound or CT examinations, to achieve early detection and warning.

Oral health interventions are an effective means of primary prevention of PC, with public health value characterized by low cost and easy promotion. Improving oral hygiene and timely treatment of PD may reduce the risk of PC (Zhou and Luo, 2019). Poor oral hygiene is a significant cause of periodontitis, and if left untreated, the condition will progress inexorably to tooth loss (Janakiram and Dye, 2020). Additionally, studies have found that patients with a low annual medical insurance reimbursement limit for periodontitis treatment (≤11 US dollars) have a significantly higher risk of PC than those with a higher reimbursement limit (adjusted hazard ratio aHR=1.35, 95% confidence interval CI = 1.03–1.79), suggesting that insufficient treatment may increase the risk of cancer through persistent systemic inflammatory responses (Li et al., 2025). Therefore, in clinical practice, oral health education can be provided to the general population to emphasize the association between PD and systemic diseases such as PC and to advocate basic oral care behaviors, such as standardized tooth brushing, flossing, and regular professional teeth cleaning (Janakiram and Dye, 2020). Active interventions can be implemented for PD patients, including mechanical treatments such as supragingival scaling, subgingival curettage, and root planing. When necessary, combined with local antibacterial agents (such as chlorhexidine and povidone-iodine) or precision therapy targeting pathogenic bacteria, to reduce the colonization of periodontal pathogens and systemic inflammatory exposure (Kowalski et al., 2022; Kurtzman et al., 2022). In addition, simultaneous interventions should be carried out for modifiable risk factors such as smoking and alcohol consumption. Smoking not only increases the risk of PD but also synergistically elevates the risk of PC by enhancing oral bacteria-mediated acetaldehyde production. Thus, quitting smoking and limiting alcohol intake can simultaneously reduce the risk of comorbidity of both diseases (Meyer et al., 2008; Ahn et al., 2012a).

Furthermore, in the field of early diagnosis and risk prediction, artificial intelligence (AI) technology shows considerable potential to optimize the efficiency and accuracy of risk screening for the PD-PC association. A systematic review focusing on AI applications in periodontal-systemic disease interactions has indicated that machine learning models integrating oral health data, clinical parameters, multi-omics profiles, and imaging data can yield notable improvements in diagnostic and predictive performance for such cross-organ disease links (Das et al., 2025). When applied appropriately, AI-based screening can serve as an efficient auxiliary tool for large-scale population screening and high-risk individual stratification in clinical settings. Therefore, in clinical practice, AI technology can be leveraged for large-scale pancreatic cancer screening in the general population, especially for individuals with periodontal disease or other known PC risk factors, to assist in clinical risk stratification, the implementation of early interventions, and the formulation of personalized treatment decisions, thereby providing valuable technical support for the clinical management of the PD-PC association. It should be noted, however, that the clinical application of such AI models still faces practical challenges, including data bias, overfitting risks, and integration with existing clinical workflows. Their performance relies on large-scale, high-quality datasets and continuous retraining and validation to ensure reliable and generalizable outcomes (Das et al., 2025).

### 4.2 Supplement and optimization of diagnostic biomarkers

Oral microbial and related molecular indicators, with their core advantages of being non-invasive and easily accessible, can serve as essential supplements to traditional diagnostic biomarkers for PC. They effectively make up for the shortcomings of insufficient sensitivity and limited specificity of existing early diagnostic methods, providing new support for the precise screening and confirmation of PC. From the perspective of single biomarkers, the detection rates of P. gingivalis and A. actinomycetemcomitans in the saliva and serum of PC patients are significantly higher than those in healthy populations (Fan et al., 2018). The serum immunoglobulin G (IgG) antibodies induced by the endotoxins of these pathogenic bacteria can persist in the systemic circulation for up to 15 years or longer (Ampomah et al., 2023). Moreover, their antibody levels are positively correlated with disease severity, suggesting that they can not only be used for disease screening but also reflect disease progression (Thanakun et al., 2016). In addition, the area under the curve (AUC) of the multi-bacteria combined detection model for diagnosing gastrointestinal cancers (including PC) can exceed 0.90, which is significantly superior to single-bacteria or traditional tumor marker detection, highlighting the diagnostic potential of combined microbial indicators (Chen et al., 2019).

In addition to microbial indicators, the differentially expressed miRNAs shared by PD and PC further enrich the diagnostic biomarker library. Studies have shown that detecting circulating miRNAs can identify high-risk individuals up to 5 years before PC confirmation, indicating its great potential for cancer screening and monitoring (Wang C. et al., 2024). Sunnetci-Akkoyunlu et al. found that molecules such as hsa-miR-155, hsa-miR-211, and hsa-miR-375 can be stably detected in serum and saliva. Among them, the AUC of hsa-miR-211 reaches 0.675, which can be used as a supplement to traditional markers to improve the detection rate of early PC and also serve as a potential combined therapeutic target for both diseases (Sunnetci-Akkoyunlu et al., 2025). An Iranian case-control study further showed that the loads of P. gingivalis and A. actinomycetemcomitans in saliva, and the copy numbers of miR-21 and miR-155 in blood, were quantified by quantitative real-time PCR. Among the constructed diagnostic models, the model combining the above two periodontal pathogenic bacteria, two circulating miRNAs, and diabetes status had the highest diagnostic efficiency, with an AUC of 0.878 (95% CI 0.802-0.955 (Hesami et al., 2025). Saliva metabolomics shows even greater performance, with an AUC of 0.993 in distinguishing PC patients from healthy controls (Sugimoto et al., 2010). Thus, saliva has certain reliability and clinical relevance as a diagnostic medium. Moreover, due to its non-invasiveness and ease of collection, saliva detection can provide a new perspective for the early identification and prognostic evaluation of PC and is also suitable for large-scale population early screening (Alkharaan et al., 2020; Ungureanu et al., 2023).

Overall, a single biomarker type is unlikely to meet the precise needs for clinical early diagnosis of PC. In the future, it is necessary to optimize further the multidimensional combined diagnostic model of “microbiome + miRNA + metabolite”. By integrating biological information from different levels, the sensitivity and specificity of diagnosis can be maximized. On this basis, combined with imaging examinations such as abdominal ultrasound and MRI, a stepwise diagnostic process of “non-invasive screening - precise confirmation” can be constructed. First, convenient combined saliva detection is used for initial screening of high-risk populations and then imaging re-examination and pathological confirmation are performed for positive cases. This model not only reduces the cost and invasiveness of large-scale screening but also effectively reduces missed diagnoses and misdiagnoses, making it especially suitable for long-term dynamic monitoring of high-risk PC populations.

### 4.3 Adjustment and innovation of treatment strategies

Targeted intervention against periodontal pathogens can serve as an auxiliary measure in the comprehensive treatment of PC, improving treatment outcomes and reversing chemoresistance. Previous mechanistic studies have shown that P. gingivalis and A. actinomycetemcomitans can metabolize and inactivate gemcitabine by expressing cytidine deaminase (CDD), or induce paclitaxel resistance by activating the Notch1 pathway (Woo et al., 2017; Xu et al., 2025). Therefore, clinical exploration can be conducted to combine targeted antibiotics with chemotherapy to eliminate pathogenic bacteria colonizing tumors and restore the sensitivity of chemotherapeutic drugs (Geller et al., 2017). Emerging regulatory approaches to flora provide a new direction for adjuvant PC treatment. Probiotic preparations (such as Veillonella and Capnocytophaga ochracea) may inhibit the growth of pathogenic bacteria, maintain oral flora balance, regulate the oral-gut-pancreas flora balance, inhibit the colonization of pathogenic bacteria, and indirectly reduce the risk of PC (Michaud et al., 2013; Ungureanu et al., 2023). Phage therapy can specifically target periodontal pathogenic bacteria such as P. gingivalis and F. nucleatum without interfering with the normal oral flora. At the same time, it can penetrate and destroy periodontal biofilms, reduce the production of drug-resistant strains, and has no apparent systemic side effects, making it suitable for patients with low immune function (Kowalski et al., 2022). In addition, cold atmospheric plasma (CAP) therapy, antimicrobial photodynamic therapy, and antimicrobial peptides can prevent the formation of oral biofilms and reduce the risk of pathogenic bacteria spreading, which can be included in comprehensive management as auxiliary treatment methods for PD (Chattopadhyay et al., 2023).

Effective periodontal tissue regeneration and anti-inflammatory therapies help alleviate PD-induced chronic systemic inflammation and reduce the potential for translocation and colonization of periodontal pathogens in pancreatic tissue, thereby laying a foundation for mitigating PC risk and supporting PC treatment efficacy. In the field of periodontal tissue regeneration and anti-inflammatory treatment, various active substances and delivery strategies have shown promising clinical potential for PD management, and their associated improvements in oral health status may have indirect implications for PC prevention and adjuvant treatment. For example, quercetin has multi-target regulatory effects. On the one hand, quercetin can activate the NRF2 signaling pathway, enhance the antioxidant capacity of periodontal ligament cells (PDLCs), reduce oxidative stress damage, and thereby alleviate alveolar bone loss in periodontitis (Wei et al., 2021). On the other hand, *in vitro* experiments have shown that quercetin can reduce oxidative stress in orofacial mesenchymal stem cells (OMSCs) and regulate OMSC osteogenic differentiation by mediating m6A modification of Per1. However, the unique dynamic and humid microenvironment of the periodontium limits the therapeutic effects of systemic and local quercetin administration. To address this problem, the study developed a biocompatible, controlled-release injectable hydrogel system composed of nanobioglass microspheres and a photocurable injectable hydrogel. This system can adapt to the special periodontal microenvironment to achieve stable loading and delivery of quercetin. It can further promote the osteogenic differentiation of OMSCs through a Per1-dependent pathway, ultimately accelerating periodontal bone repair (Zhu et al., 2024). In addition, silver nanoparticles (AgNPs) have emerged as a promising therapeutic approach for the management of periodontal disease due to their potent antibacterial activity and ability to promote tissue regeneration. A study using a ligature-induced rat periodontitis model showed that, compared with the untreated and standard treatment groups, the AgNP patch treatment group demonstrated significant improvements in gingival and periodontal tissue repair and reduced inflammation. Among them, the high-dose AgNP patch (1000 mg/kg) treatment group showed nearly complete tissue repair effect (Barik et al., 2025). This local anti-inflammatory and tissue-repairing effect of AgNPs may effectively reduce the systemic inflammatory load induced by PD and inhibit the proliferation and dissemination of periodontal pathogenic bacteria, thereby indirectly mitigating the potential risk posed by these pathogens in mediating PC development and chemoresistance. The above studies provide diverse and effective strategies for clinical PD treatment, including targeted regulation of active substances and local interventions with new materials. The effective clinical implementation of these strategies may help optimize oral health, mitigate systemic inflammatory responses and reduce pathogenic bacterial translocation associated with PD, thereby offering a supportive oral health management approach for PC prevention and enhanced chemotherapy efficacy. However, existing treatments are difficult to achieve complete tissue regeneration, and the potential indirect effects of these PD therapeutic strategies on PC remain to be verified by more in-depth clinical and experimental studies. In the future, it will be necessary to develop further comprehensive therapies that integrate microbiomics, epigenetics, and immune regulation, with the dual exploratory goal of improving periodontal health and reducing the potential impact of PD on PC occurrence and progression.

### 4.4 Cross-disciplinary collaboration: multidisciplinary team management of PD and PC

Given the potential correlation and multi-dimensional pathogenic mechanisms between PD and PC, establishing an integrated multidisciplinary team (MDT) collaboration model involving “Dentistry - Oncology - Gastroenterology - Microbiology - Radiology” is a key practical direction for optimizing the clinical management of PC and improving patient prognosis. From a clinical perspective, PD-related microbial imbalance and chronic inflammation may not only increase the risk of PC but also affect treatment outcomes by inducing chemoresistance and exacerbating immunosuppression in the tumor microenvironment. A single discipline is complex and cannot fully cover the end-to-end needs of “risk screening - treatment intervention - prognostic monitoring”, making MDT an inevitable choice. In collaborative practice, dentistry should be deeply integrated into the management of high-risk PC populations. Periodontal health assessment, including indicators such as periodontitis diagnosis, probing depth, clinical attachment loss, and number of missing teeth, should be included in the routine screening process (El Ayachi et al., 2025). For high-risk individuals with moderate to severe periodontitis, standardized interventions such as supragingival scaling, subgingival curettage, and targeted antibacterial therapy should be implemented simultaneously (Kowalski et al., 2022). Personalized PC screening plans should be formulated in conjunction with oncology departments, including regular abdominal imaging, to lay the foundation for early warning and intervention by reducing the burden of periodontal pathogenic bacteria and systemic inflammation. During the treatment phase, it is necessary to establish a cross-disciplinary joint monitoring mechanism. Given that P. gingivalis and A. actinomycetemcomitans can metabolize and inactivate gemcitabine by expressing CDD or induce paclitaxel resistance by activating the Notch1 pathway (Woo et al., 2017; Xu et al., 2025), baseline oral health assessment and necessary interventions should be completed before chemotherapy. Periodic re-examinations of periodontal status should be conducted during treatment. If acute inflammatory episodes occur, combined anti-infective treatment should be initiated promptly to avoid a reciprocal worsening cascade of “chemotherapy-induced damage - periodontal inflammation - treatment resistance” (Sunnetci-Akkoyunlu et al., 2025). At the same time, microbiology departments can participate in the development of precise therapies targeting periodontal pathogenic bacteria, such as phage therapy and probiotic preparations. Radiology and AI departments can improve the accuracy of risk stratification and prognostic prediction by integrating oral microbial data, serum miRNA expression profiles, and tumor imaging features (Das et al., 2025). Currently, MDT still faces challenges, including unclear mechanisms of association, a lack of standardized clinical processes, and insufficient patient awareness of the association between oral health and PC. In the future, multicenter prospective studies are needed to verify further the causal relationship, such as the “Expert Consensus on Oral Health Management for Pancreatic Cancer Patients”. At the same time, cross-disciplinary science popularization should be strengthened to enhance compliance with doctor-patient collaboration and, ultimately, seamlessly integrate oral health management into the entire cycle of PC prevention and treatment, providing innovative, practical support to improve patient prognosis.

## 5 Conclusion

This review synthesizes the epidemiological evidence, multi-dimensional mechanisms, and clinical implications of the PD-PC association. Epidemiological studies suggest that moderate-to-severe PD, particularly the gingivitis-periodontitis subtype, may be linked to increased PC incidence and mortality, with Porphyromonas gingivalis and Aggregatibacter actinomycetemcomitans as key drivers and prominent associations in middle-aged and elderly individuals, males, and those with comorbidities. Mechanistically, PD may promote PC through interconnected pathways: pathogenic colonization and precancerous lesion induction; activation of TLR4/NF-κB and Wnt/β-catenin inflammatory signaling; immune dysregulation and tumor escape; microbiota imbalance with carcinogenic metabolite accumulation; and induction of gemcitabine/paclitaxel resistance. Clinically, this association enables actionable strategies: integrating periodontal health assessment into PC risk screening, developing non-invasive biomarkers (oral microbiota, miRNAs, saliva metabolites), optimizing therapies (targeted antibiotics, probiotics, phage therapy), and establishing a dentistry-oncology-gastroenterology MDT model. Future prospective studies are needed to confirm causality and standardize clinical protocols, leveraging oral health management as a cost-effective approach to improve PC prevention and prognosis.
